# Supplementary material for: Identification of Freezing-Responsive microRNAs and Their Targets in Chinese Jujube by Small RNA and Degradome Sequencing
Source: Int J Mol Sci. 2026 Sep 4;27(17):7912. doi: 10.3390/ijms27177912 (PMC13566890; doi:10.3390/ijms27177912)
Supplement: Supplementary file 1 [file ijms-27-07912-s001.zip › Supplementary Material S2/novel_miR_80.pdf]

Provisional ID	:	novel_miR_80
Score total	:	0.6
Score for star read(s)	:	-1.3
Score for read counts	:	0
Score for mfe	:	0.3
Score for randfold	:	1.6
Score for cons. seed	:	
Total read count	:	93
Mature read count	:	93
Loop read count	:	0
Star read count	:	0

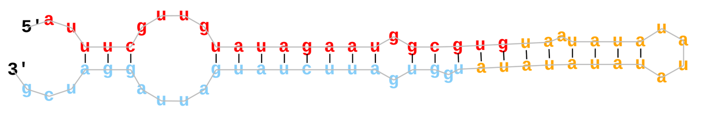

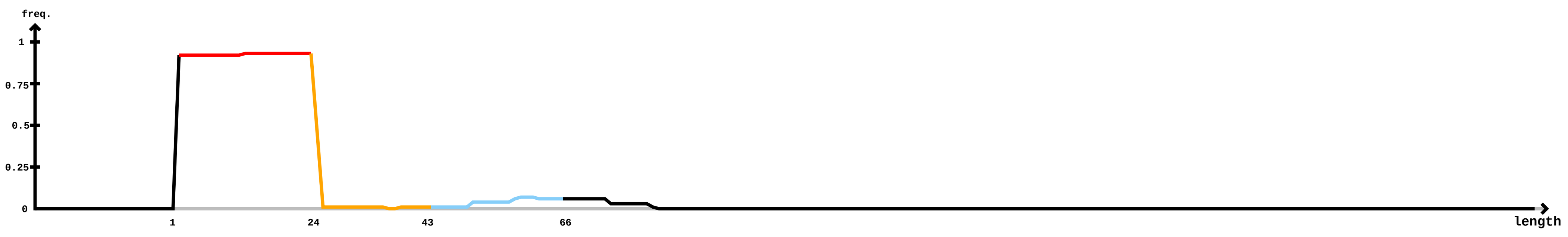

Mature Star

```
5'- gguagagaugguagacugguugauuucguuguauagaauggcguguaauauauauauauauauauaauggugauucuaugauuaggaucguccacauacaaacuaaaagaaaaagccgaugccuuuuuggaaauaaaaauguaaaaagcgucgguuuuucucucaccaacguuuuuuagaauuuuauuuuuuuaaaaauaucaguuuuuucuugaaaagcgucgguuuuucucucacuaacauuuuuuagaau -3'    exp
    (((.((((..(((...(((.((((((.....((((((((.(((((((.((((....)))))))))).)).))))))))....)))))).)))..))).......((((((((((.((((..(((..((((((((((.(.((((((((((.(((.........))).))))))))))).).))))))))))..)))..)))).))))))))))(((((((....)))))))))))))))...............    reads   mm   sample
    ......................Cuuucguuguauagaauggcgug..................................................................................................................................................................................................    1       1    LCK-1
    ......................auuucguuguauagaauggcgug..................................................................................................................................................................................................    46      0    LCK-1
    ......................auuucguuguauagaauggcgug..................................................................................................................................................................................................    46      0    LCK-2
    .................................uagaauggcguguaauauauauau......................................................................................................................................................................................    1       0    LT-2
    .............................................................auauaGggugauucuaugauuag...........................................................................................................................................................    1       1    LT-1
    .......................................................................ucuaugauuaggaucguccacau..................................................................................................................................................    1       0    LT-1
    .......................................................................ucuaugauuaggaucguUcacau..................................................................................................................................................    2       1    LT-1
    ..............................................................................uuaggaucguccacauacaaacu...........................................................................................................................................    2       0    JCK-2
    ...............................................................................uaggaucguccacauacaaacua..........................................................................................................................................    1       0    LT-3
```
